# Quantum and classical dynamics comparison: A way for selecting the vector potential across diverse frames

**DOI:** 10.1016/j.heliyon.2025.e42718

**Published:** 2025-02-21

**Authors:** Maryam Pakpour, Amir H. Darooneh, Mohammad Mahmoudi

**Affiliations:** a Department of Physics, University of Zanjan, University Blvd., 45371-38791, Zanjan, Iran; b Pasargad Institute for Advanced Innovative Solutions (PIAIS), Tehran, 19916-33361, Iran

**Keywords:** Gauge transformation, Intense laser field, Krammers-Henneberger transformation

## Abstract

This study explores the quantum dynamics of a free electron subjected to an intense laser field, considering various gauges within and beyond the dipole approximation. The quantum path, as dictated by the Ehrenfest theorem, is systematically computed. Notably, it is revealed that the quantum path aligns with the classical trajectory in the laboratory frame exclusively for a specific vector potential. Additionally, the quantum paths derived from alternative gauges exhibit notable agreement with the classical trajectories within their respective moving frames. This novel approach introduces a method for selecting the appropriate vector potential in the laboratory frame.

## Highlights

• The quantum dynamics of a free electron in an intense laser field is investigated.
• The quantum proposed path is computed within dipole approximation and beyond it.
• We provide a method for selecting the vector potential across diverse frames.

## Graphical abstract

**Figure:**
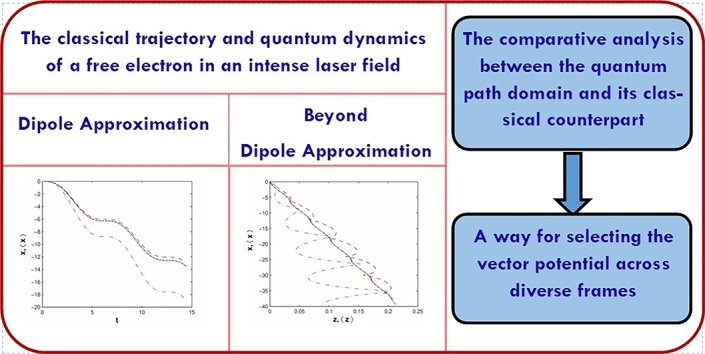

## 1 Introduction

The recognition of the symmetry of the quantum evolution equation under gauge transformation dates back to Fock, who initially observed it while investigating the relativistic wave equation for spinless particles [1]. Subsequently, Weyl emphasized the significance of gauge invariance as a guiding principle in constructing a quantum theory for charged particles [1], [2]. Pauli further popularized the concept of gauge invariance in quantum mechanics [3]. In the 1950s, the principle of gauge invariance extended to the quantum theory of strong interactions [4], [5], [6], and later found application in the quantum theory of (electro)weak interactions [7], [8].

Gauge invariance serves as a fundamental principle in the quantum theory of matter, offering numerous advantages such as charge conservation, the existence of massless photons, and phenomena like superconductivity phase transitions, all stemming from gauge symmetry or its breaking [7], [8]. Despite these advantages, it is crucial to note that the time-dependent Schrodinger/Dirac equation is gauge-dependent, particularly concerning time-dependent electromagnetic potentials [9], [10], [11], [12], [13], [14], [15], [16], [17], [18], [19]. The choice of different gauges leads to disparities in results, as evidenced in photodetachment outcomes [16], [19] and simulation results for photodesorption yield from metallic surfaces using the dipole approximation with different laser-matter interaction terms in the Hamiltonian [17].

The existence of non-Volkov solutions for Dirac and Klein-Gordon equations, describing the motion of charged particles in a plane electromagnetic wave, directly correlates with the adoption of different gauges [18]. Several attempts have been made to address this issue in the quantum theory of matter. For instance, adding a complete time-derivative term to the Lagrangian renders the Hamiltonian gauge-invariant but introduces path dependence [12]. Kobe et al. propose replacing the time derivative of the quantum power operator with the Hamiltonian for the energy operator [13]. Aharanov et al. address this challenge by introducing an acceleration-dependent term in the classical Lagrangian or Hamiltonian [14].

The interaction between high-power lasers and matter, as well as the dynamics of free electrons in this context, has undergone extensive investigation [20], [21], [22]. A comparative analysis between classical and quantum approaches proves to be a valuable tool for deriving diverse physical outcomes [23], [24], [25]. Bohmian mechanics has been employed in the realm of intense laser-atom physics, where the motion of an atomic electron in an intense laser field is determined by the Bohm-Newton equation. It has been demonstrated that semiclassical trajectory methods in intense laser-atom physics treat the motion of the ionized electron using classical mechanics, transitioning to quantum mechanics prior to ionization [26].

This study focuses on the classical and quantum dynamics of an ionized free electron in an intense laser field, introducing a novel method for selecting the vector potential in the laboratory frame. Additionally, we assert that gauge transformations reveal the vector potential in distinct moving frames. The quantum dynamics of the free electron are computed in frames corresponding to different gauges, revealing a substantial agreement with the classical trajectory. A free electron in an external field exemplifies a hybrid quantum-classical system [27] and can indeed be approached using a quantum-classical density matrix [28]. It is important to note that in statistical mechanics, when correlations between the classical and quantum components of a system are negligible, and these components are initially in pure states, the evolution can be described by the Schrödinger equation for the quantum part [29]. Note that the solution of the time-dependent Schrödinger/Dirac equation depends on the gauge due to the time-dependent electromagnetic vector potentials. However, the gauge-dependence of electron dynamics in laser fields is analogous to the concept of frame-dependence of classical trajectories in which the trajectory measured in the laboratory frame can appear different from the perspective of moving observers. This does not imply that the results are fundamentally different; rather, they are related through transformations between reference frames.

We structure our paper as follows. In the subsequent section, we address the challenge posed by a free charged particle subjected to an external electromagnetic wave. The third section is dedicated to elucidating the Generalized Henneberger method [31], which provides an analytical solution for the interaction of a charged particle with an electromagnetic wave beyond the dipole approximation. The fourth section provides an account of our findings concerning the solutions to the aforementioned problem in two distinct gauges, each characterized by a constant. Lastly, we present a summary of our results in the concluding section.

## 2 Classical motion

To consider the interaction of a free electron with electromagnetic intense laser field, we assume that the free electron has a mass *m* and charge −*e* and we also model the laser field by an electromagnetic field **E** in the space time coordinate (r,t),

(1) $$E=\overset{ˆ}{x}E_{0}\sin(\omega \eta )$$

where η=t−z/c, the propagation direction is along *z*-axis. $E_{0}$ is a constant amplitude and *ω* is the frequency of laser field.

Two cases of the free electron field interactions will be investigated, what follows namely, with dipole approximation (DA) and beyond it. In dipole approximation, kz≪1, the magnetic component of electromagnet field is neglected. The equation of motion of a free electron in laser field can be written as,

(2) $$m\overset{¨}{x}=-eE_{0}\sin(\omega t).$$

Then,

(3) $$x(t)=\frac{eE_{0}}{m\omega^{2}}\sin(\omega t)-\frac{eE_{0}}{m\omega }t.$$

It is assumed that the electron initially (at t=0) is at the origin of coordinates and at the rest condition.

In atomic and molecular physics, it is convenient to use the elementary charge, *e*, as the unit of charge, and the electron mass, $m_{e}$, as the unit of mass. Another quantity that appears all over in quantum physics is Planck's constant divided by 2*π*, which has dimensions $ML^{2}T^{-1}$; so it is convenient to choose units of length and time such that ħ=1 and $4\pi \epsilon_{0}=1$. It is worth to note that $E_{0}=1\;a.u.$ is equivalent to an electric field strength of about $5.4\times 10^{9}V/m$. This is much less than the QED critical field strength $E_{crit.}=1.3\times 10^{16}V/m$ at which a static electric field would spontaneously break down into electron-positron pairs.

Beyond dipole approximation (BDA), the magnetic component of the Lorentz force on the free electron becomes comparable to the electric component when the velocity approaches to the speed of light. As we know, such situation happens under conditions of interaction with a high intensity laser field.

The nonrelativistic equation of motion for a classical charged particle in a linearly polarized plane laser field, Eq. (1) reads,

(4) $$m\overset{˙}{v}=-e(E+\frac{v}{c}\times B),$$

where the laser magnetic field is,

(5) $$B=\overset{ˆ}{y}E_{0}\sin(\omega \eta )$$

It is easy to understand for any function Q(η), we have,

(6) $$\frac{dQ}{dt}=\frac{d\eta }{dt}\frac{dQ}{d\eta }=(1-\frac{v_{z}}{c})\frac{dQ}{d\eta }.$$

The Eq. (4) transforms into the following set of equations.

(7) $$m\frac{dv_{x}}{d\eta }=-eE_{0}\sin(\omega \eta ),$$

(8) $$m\frac{dv_{y}}{d\eta }=0,$$

(9) $$m\frac{dv_{z}}{d\eta }=-\frac{e}{c}v_{x}E_{0}\sin(\omega \eta ).$$

For an electron at the origin of coordinates at t=0, under initially the rest condition the solution of above equations can be written as,

(10) $$x(\eta )=Kc\int_{0}^{\eta }\frac{\sin^{2}(\omega \eta )d\eta }{{\left[1-K^{2}\sin^{4}(\omega \eta /2)\right]}^{1/2}},$$

(11) $$z(\eta )=c\left[\int_{0}^{\eta }\frac{d\eta }{{\left[1-K^{2}\sin^{4}(\omega \eta /2)\right]}^{1/2}}-\eta \right],$$

where $K=2eE_{0}/m\omega c$ in atomic unit.

In the following sections we use the above solutions for comparison between classical and quantum behavior of an electron.

## 3 Quantum description

We are interested in discribing the quantum behavior of a free electron in an intense laser field. In general, the Hamiltonian of such system can be given as,

(12) $$H=\frac{{\left(p+eA/c\right)}^{2}}{2m}=\frac{p^{2}}{2m}+\frac{e}{mc}A\cdot p+\frac{e^{2}A^{2}}{2mc^{2}}=H_{f}+H_{i},$$

where $H_{f}=p^{2}/2m$ represents kinetic energy of the free electron, $H_{i}$ describes its interaction with the radiation field and *c* is the speed of light in vacuum. Here we employ the Coulomb gauge (∇⋅A=0).

The dynamics of the system are governed by Schrödinger's equation,

(13) $$H\Psi =i\hbar \frac{\partial \Psi }{\partial t}.$$

In the next step we solve the Schrödinger's equation for *ψ*, however, it is easier to solve Schrödinger's equation in the Krammers-Henneberger (KH) frame. This is a non-inertial reference frame in which the well-known quiver motion of the free electron in the laser field may be eliminated (exactly within the dipole approximation and approximately without it). The wave function in the KH frame is related to its laboratory counterpart by the following unitary transformation

(14) $$\Psi_{KH}(r,t)=U\Psi (r,t);\;U\equiv \exp(\frac{i}{\hbar }\int H_{i}(r,t^{\prime })dt^{\prime }),$$

and the Schrödinger equation becomes

(15) $$UH_{f}U^{†}\Psi_{KH}=i\hbar \frac{\partial \Psi_{KH}}{\partial t}.$$

In the Coulomb gauge, the two terms making up $H_{i}$ commute and then the transformation may be written as $U=U_{1}U_{2}$ where,

(16) $$U_{1}=\exp(\alpha \cdot \nabla );\;U_{2}=\exp(D),$$

and

(17) $$\alpha =\frac{e}{mc}\int A(r,t^{\prime })dt^{\prime },$$

(18) $$D=\frac{ie^{2}}{2mc^{2}\hbar }\int A^{2}(r,t^{\prime })dt^{\prime }.$$

On the other hand, in general we have,

(19) $$UH_{f}U^{†}\approx H_{f}+H^{\prime };\;H^{\prime }=\left(\frac{i\hbar }{m}\right)\frac{\partial D}{\partial z}p_{z}.$$

$H^{\prime }$ contains the operator $p_{z}$ and leads a simple translation in the *z* direction. Then Eq. (15) can be written as,

(20) $$i\hbar \frac{\partial \Psi_{KH}}{\partial t}\approx (H_{f}+H^{\prime })\Psi_{KH}.$$

The second transformation is introduced by,

(21) $$\Phi_{KH}=\Omega \Psi_{KH};\;\Omega \equiv \exp(\frac{i}{\hbar }\int H^{\prime }dt^{\prime }).$$

With applying the second transformation by Ω, the Schrödinger equation becomes,

(22) $$i\hbar \frac{\partial \Phi_{KH}}{\partial t}\approx -\frac{\hbar^{2}}{2m}\nabla^{2}\Phi_{KH},$$

which is a noninteracting particle Schrödinger equation with defined solution. Then the analytical solution of Eq. (13) can be written as [31],

(23) $$\Psi =U_{2}^{†}U_{1}^{†}\Omega^{†}\Phi_{KH}(x,y,z;t)=U_{2}^{†}U_{1}^{†}\Phi_{KH}(x,y,z-\beta ;t)=U_{2}^{†}\Phi_{KH}(x-\alpha ,y,z-\beta ;t)$$

where Ω is rewritten as Ω=exp⁡(β⋅∇), with

(24) $$\beta \equiv \overset{ˆ}{z}\left(\frac{i\hbar }{m}\right)\int \frac{\partial D}{\partial z}dt^{\prime }=-\overset{ˆ}{z}\left(\frac{i\hbar }{mc}\right)D.$$

The details of calculation will be discussed in the next section. Note that, in the dipole approximation, ∂D/∂z=0 implies Ω=1 and hence, β=0.

### 3.1 Dipole approximation

In dipole approximation, kz≪1, the electromagnetic wave is assumed as dynamical electric field which is obtained by the following vector potential (Rau's gauge [16])

(25) $$A(t)=\overset{ˆ}{x}\frac{cE_{0}}{\omega }[\cos(\omega t)-1].$$

Presence of the constant term in Eq. (25) does not affect the observable electromagnetic fields and has the advantage of making **A** well-behaved in the zero-frequency limit [16]. The simple calculations leads to

(26) $$\alpha =\overset{ˆ}{x}\frac{eE_{0}}{m\omega^{2}}\sin(\omega t),$$

(27) $$D=\frac{ı(eE_{0}^{2})}{8m\omega^{3}\hbar }[\sin(2\omega t)+2\omega t],$$

(28) β=0,

(29) $$U_{2}=\exp(D).$$

For a realistic representation of localized free electron (classical free electron), a Gaussian wave packet moving in *z* direction, with nonzero extension along *x*, seems more appropriate. Our choice is the 2*D* wave packet [30]

(30) $$\Phi_{KH}=\frac{1}{a\sqrt{2\pi }(1+it/\tau )}\exp\left[\frac{i\tau }{t}\left(\frac{x^{2}+z^{2}}{4a^{2}}\right)\right]\times \exp\left\{-\frac{i\tau /4a^{2}t}{1+it/\tau }\left[x^{2}+{\left(z-\frac{p_{0}}{m}t\right)}^{2}\right]\right\},$$

where $\tau =2ma^{2}/\hbar$, *a* represents reasonable size of the wave packet, and $p_{0}$ stands for the initial momentum of the free electron. Assuming, it is born from an atom, possibly by ionization, a size for the free electron may be taken as a∼Δx∼Δz. This wave packet leads to a probability distribution which describes a point particle in the a→0 limit. In other words, it turns out to be zero everywhere except on the classical particle trajectory [30]. Then the solution of Schrodinger equation for a free electron in a laser field, in DA is given by [31]

(31) $$\psi^{\left(DA\right)}=\frac{\exp(-D)}{a\sqrt{2\pi }(1+it/\tau )}\exp[\frac{i\tau }{t}(\frac{{\overset{¯}{x}}^{2}+z^{2}}{4a^{2}})]\times \exp\{-\frac{i\tau /4a^{2}t}{1+it/\tau }\;[{\overset{¯}{x}}^{2}+{\left(z-\frac{p_{0}t}{m}\right)}^{2}]\}.$$

Where $\overset{¯}{x}=x-\alpha$.

The classical limit of quantum mechanics is usually discussed in terms of Ehrenfest theorem, which establishes a connection between the quantum mechanical evolution of expectation values and the corresponding classical equations of motion below the Ehrenfest time. The Ehrenfest time is a concept in quantum mechanics that describes the time scale over which quantum dynamics closely resemble classical dynamics for a quantum system. In non-chaotic systems, the Ehrenfest time, growing inversely with *ħ*, is typically long enough for classical dynamics to provide a reliable approximation over practical time scales. The Ehrenfest theorem has been also generalized for bipartite quantum-classical hybrid systems [32]. According to this theorem, for a sufficiently localized wave packet, the expectation value of position in quantum state will follow a classical trajectory [33].

The expectation value of *x* can be calculated by

(32) $$<x>=<\psi^{\left(DA\right)}|\;x\;|\psi^{\left(DA\right)}>=\int_{-\infty }^{+\infty }\int_{-\infty }^{+\infty }\psi_{\left(x,z;t\right)}^{⁎(DA)}\;x\;\psi_{\left(x,z;t\right)}^{\left(DA\right)}\;dx\;dz.$$

**Figure 1:**
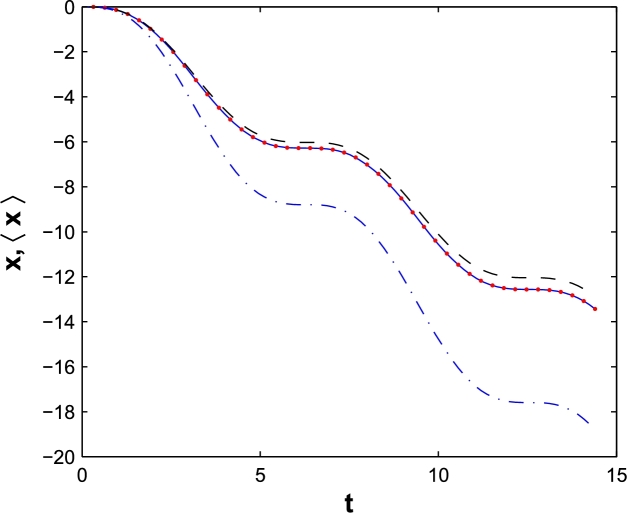
The classical trajectory (solid) of an electron in an intense laser field with *E*_0_ = 1*a.u*. and *ω* = 1 *a.u*. in dipole approximation. The quantum proposed domain for a *a* = 10 *a.u*. (Red points), *a* = 50 *a.u*. (Dashed), a=100 a.u. (Dash-dot). The results are plotted in the laboratory frame.

The quantum domain of classical trajectory of a free electron in a laser field and dipole approximation is plotted in Fig. 1. The field amplitude $E_{0}=1\;a.u$. and frequency ω=1a.u. are the same for the classic and quantum cases. The width of wave packet is a=10a.u. (Red points), a=50a.u. (Dashed) and a=100a.u. (Dash-dot).

An investigation on Fig. 1 shows that the classical trajectory is in good agreement with the quantum domain, proposed by Ehrenfest theorem. For a narrow wave packet, i.e. a=10a.u., both trajectories are exactly the same, but by increasing the width of wave packet, as expected, the quantum domain path is distinguished from classical one.

### 3.2 Beyond dipole approximation (BDA)

Recall that in dipole approximation, the effect of magnetic component of a laser field on free electron dynamics, is ignored [34], [35]. In high intensity laser field, the velocity of free electron approaches the velocity of light in vacuum and the effect of magnetic component of Lorentz force on the free electron dynamics is more important.

Now, we consider the condition of beyond dipole approximation (BDA). In this regime, the corresponding vector potential can be written as

(33) $$A=\overset{ˆ}{x}\frac{cE_{0}}{\omega }[\cos(\omega \eta )-1].$$

Using it we get the following BDA counterparts for Eqs. (26)− (29)

(34) $$\alpha =\overset{ˆ}{x}\frac{eE_{0}}{m\omega^{2}}\left[\sin(\omega \eta )-\omega \eta \right],$$

(35) $$D=\frac{i}{\hbar }\frac{{\left(eE_{0}\right)}^{2}}{8m\omega^{3}}\left[\sin(2\omega \eta )-8\sin(\omega \eta )+6\omega \eta \right],$$

(36) $$\beta =-\overset{ˆ}{z}\left(\frac{i\hbar }{mc}\right)D,$$

(37) $$U_{2}=\exp(D).$$

The Eqs. (33)− (35) are derived from Eqs. (25)− (27), respectively, by replacing *ωt* with *ωη*, as in Ref. [16]. Eventually, the following expression for wave function, $\Psi^{BDA}$, in the laboratory frame is,

(38) $$\Psi^{\left(BDA\right)}=\frac{\exp(-D)}{a\sqrt{2\pi }(1+it/\tau )}\exp\left[\frac{i\tau }{t}\left(\frac{{\overset{¯}{x}}^{2}+{\overset{¯}{z}}^{2}}{4a^{2}}\right)\right]\times \exp\left\{-\frac{i\tau /4a^{2}t}{1+it/\tau }\left[{\overset{¯}{x}}^{2}+{\left(\overset{¯}{z}-\frac{p_{0}}{m}t\right)}^{2}\right]\right\},$$

with

(39) $$\overset{¯}{x}=x-\alpha \;and\;\overset{¯}{z}=z-\beta .$$

The expectation value of displacement vector **r** is given by

(40) $$<r>=<\psi^{\left(BDA\right)}|\;r\;|\psi^{\left(BDA\right)}>=\int_{-\infty }^{+\infty }\int_{-\infty }^{+\infty }\psi_{\left(x,z;t\right)}^{⁎(BDA)}\;r\;\psi_{\left(x,z;t\right)}^{\left(BDA\right)}\;dx\;dz.$$

**Figure 2:**
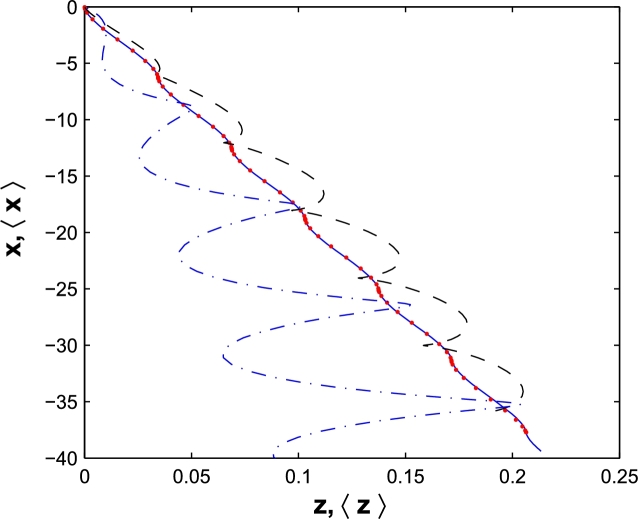
The classical trajectory (solid) of an electron in an intense laser field with *E*_0_ = 1*a.u*. and *ω* = 1 *a.u*. beyond dipole approximation (BDA). The quantum proposed domain for a *a* = 10 *a.u*. (Red points), *a* = 50 *a.u*. (Dashed), *a* = 100 *a.u*. (Dash-dot). The results are plotted in the laboratory frame.

In Fig. 2 we plot the expectation value of *x* versus *z*. The common parameters are same as classical trajectory, beyond dipole approximation. The width of wave packet is a=10a.u. (Red points), 50a.u. (Dashed), 100a.u. (Dash-dot). Although, the quantum path for broaden wave packet is different than classical trajectory, but for localized wave packet, i.e., a=10a.u. the exact overlapping is obtained. It is difficult to detect any appreciable difference between the classical trajectory and quantum domain for a more localized wave packet.

## 4 Results and discussion

Let us consider another gauge for the vector potential, describing electromagnetic field of Eq. (1). We consider following transformation for the vector potential,

(41) $$A\to A_{g}=A+A_{0},$$

where $A_{0}=\nabla \chi$ is a constant vector and *χ* shows the gauge function. In a special case, in which the vector $A_{0}$ is parallel with **A**, the electromagnetic field, Eq. (1), are invariance. Such gauge transformation changes the definition of kinetic momentum. We would like to find the corresponding proper gauge which satisfies the initial condition of free electron in laboratory frame. The main question in this paper is that, which gauge function is appropriate for a laboratory frame among the infinite number of them? The answer to this question then introduces a new approach for choosing a suitable gauge function. We choose a gauge function that the results of quantum mechanics is in good agreement by the classical one in laboratory frame.

The Hamiltonian of a such transformation reads

(42) $$H_{g}=\frac{1}{2m}{\left[p+\frac{e}{c}(A+A_{0})\right]}^{2}=H+H^{\prime },$$

where

(43) $$H=\frac{1}{2m}{\left(p+\frac{e}{c}A\right)}^{2}$$

(44) $$H^{\prime }=\frac{e}{mc}p.A_{0}+\frac{e^{2}}{mc^{2}}A\cdot A_{0}+\frac{e^{2}}{2mc^{2}}A_{0}^{2}.$$

*H* is the Hamiltonian of system before transformation. The next step would be to solve Schrödinger's equation

(45) $$H_{g}\Psi_{g}=i\hbar \frac{\partial \Psi_{g}}{\partial t},$$

for determinatoin of $\Psi_{g}$. Let us introduce the new wave function

(46) $$\Phi_{g}(r,t)=U_{g}\Psi_{g}(r,t),\;U_{g}\equiv \exp(\frac{i}{\hbar }\int H^{\prime }(r,t^{\prime })dt^{\prime }).$$

Three terms in $H^{\prime }$ commute and unitary transformation $U_{g}$ can be written as

(47) $$U_{g}=U_{g1}U_{g2}U_{g3},$$

where

(48) $$U_{g1}=\exp(\frac{ie^{2}A_{0}^{2}\;t}{2mc^{2}\hbar }),$$

(49) $$U_{g2}=\exp(\alpha_{g}\cdot \nabla ),$$

(50) $$U_{g3}=\exp(\frac{ieA_{0}\cdot \alpha }{\hbar c}),$$

(51) $$\alpha_{g}=\frac{eA_{0}}{mc}t.$$

The parameter *α* was introduced in Eq. (17). It is easy to see that $U_{g1}$ and $U_{g3}$ show the simple phases, while $U_{g2}$ means translation in the *x* direction. An investigation on Eqs. (49) and (51) shows that the new frame moves with a constant velocity in the *x* direction, with respect to the old one.

By applying a second translation, $\Omega_{g}$, in *z* direction (see appendix) the Schrödinger's equation becomes

(52) $$i\hbar \frac{\partial \phi_{g}}{\partial t}\approx H\phi_{g}.$$

Then the wave Packet is given by

(53) $$\psi_{g}(x,y,z;t)=U_{g3}^{†}U_{g2}^{†}U_{g1}^{†}\Omega_{g}^{†}\Phi_{g}(x,y,z;t)=U_{g3}^{†}U_{g2}^{†}U_{g1}^{†}\Phi_{g}(x,y,z-\beta_{g};t)=U_{g3}^{†}U_{g1}^{†}\phi_{g}(x-\alpha_{g},y,z-\beta_{g};t),$$

where

(54) $$\beta_{g}=-\frac{eA_{0}}{mc}\int \frac{\partial \alpha }{\partial z}dt^{\prime }.$$

An investigation on Eqs. (53)-(54) shows that a gauge transformation, in general, leads to a translation in x−z plane. The moving frame, in dipole approximation, has a constant velocity only in *x* direction, while BDA, it has also an oscillatory term in *z* direction, with respect to the laboratory frame (a frame defined by choosing the correct gauge function).

Let us consider a special case, in which **A** satisfy Eq. (33) and $A_{0}=\overset{ˆ}{x}\frac{cE_{0}}{\omega }=\nabla (\frac{cE_{0}}{\omega }x)$. Then we can calculate the following counterparts,

(55) $$A_{g}=\overset{ˆ}{x}\frac{cE_{0}}{\omega }[\cos(\omega \eta )],$$

(56) $$\alpha_{g}=\overset{ˆ}{x}\frac{eE_{0}}{m\omega }t,$$

(57) $$\beta_{g}=\overset{ˆ}{z}\frac{{\left(eE_{0}\right)}^{2}}{m^{2}c\omega^{3}}\sin(\omega \eta ),$$

(58) $$U_{g1}=\exp(\frac{iE_{0}^{2}\;t}{2m\omega^{2}\hbar }),$$

(59) $$U_{g3}=\exp(D_{g}),$$

(60) $$D_{g}=\frac{ı\;ecE_{0}^{2}}{\hbar \omega^{2}}[\cos(\omega \eta )-1].$$

Therefore the wave packet of a free electron according to such gauge, is determined by Eqs. (46)-(60). Note that for dipole approximation, ∂α/∂z=0 implies $\beta_{g}=0$.

**Figure 3:**
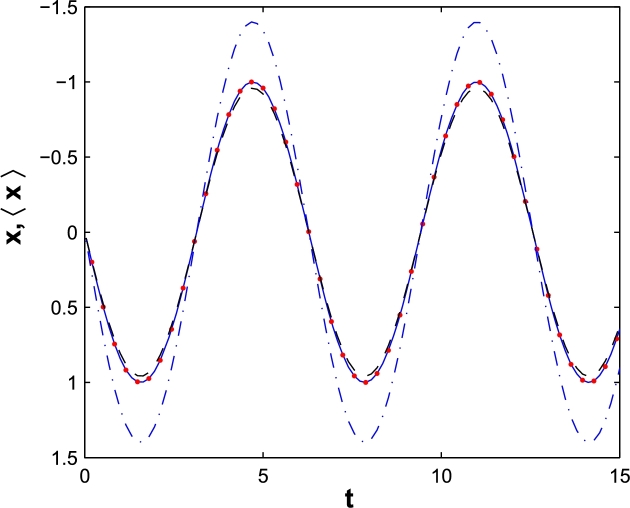
The classical trajectory (solid) of a free electron in DA, in moving coordinate and the quantum proposed domain for a=10 a.u. (Red points), a=50 a.u. (Dashed), a=100 a.u. (Dash-dot).

We are interested in calculating the expectation value of **r** in moving frame and compare the results with classical trajectory. In first step we consider dipole approximation and in Fig. 3, we plot the expectation value of *x* versus *t*. The parameters are same as Fig. 1. The solid line in Fig. 3 shows the classical trajectory of electron in moving frame. It is in good agreement with trajectory determined by Ehrenfest theorem, at least for much more localized wave packets, i.e. a=10a.u. (Red points) and a=50a.u. (Dashed). Then by choosing the second vector potential, $A_{g}$, the trajectory proposed by Ehrenfest theorem dose not follow the classical one, in laboratory frame.

**Figure 4:**
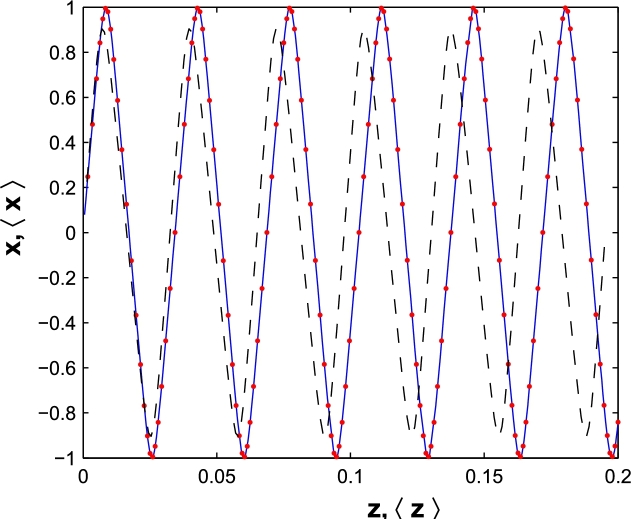
The classical trajectory (Solid) of a free electron in BDA, in moving coordinate and the quantum proposed domain for a=10 a.u. (Red points), a=50 a.u. (Dashed).

In second step, we consider BDA and plot the expectation value of *x* versus expectation value of *z* in Fig. 4. The classical trajectory of a free electron in a moving frame has been shown with solid line. The quantum domain proposed by $A_{g}$ follows the classical one, at least in small size of *a*. Note that for a=10a.u. (Red points) the classical trajectory in moving frame is exactly coinciding with the quantum path domain proposed by Ehrenfest theorem. Then by choosing a second gauge function, the results are obtained in a moving frame. Moreover, the suitable gauge function obtain the quantum mechanical results in a laboratory frame. This can be chosen by comparing the quantum mechanical results, in classical limit, with the classical experimental results. Our results introduce a suitable gauge function for working in a laboratory frame. By choosing an other gauge function, the obtained results are corresponding to a moving observation frame.

## 5 Conclusion

In conclusion, our investigation has demonstrated that the existence of distinct quantum path domains arises from the selection of different gauge functions. The comparative analysis between the quantum path domain and its classical counterpart provides valuable insights, enabling the identification of a suitable gauge function for laboratory frame applications. Furthermore, we have established that different gauge functions in quantum mechanics align with distinct observation frames in motion. This underscores the significance of gauge function selection not only in shaping quantum paths but also in determining the associated observation perspectives in the quantum realm.

## CRediT authorship contribution statement

**Maryam Pakpour:** Writing – original draft, Software, Methodology, Conceptualization. **Amir H. Darooneh:** Writing – original draft, Software, Conceptualization. **Mohammad Mahmoudi:** Writing – review & editing, Software, Project administration, Methodology, Conceptualization.

## Declaration of Competing Interest

The authors declare that they have no known competing financial interests or personal relationships that could have appeared to influence the work reported in this paper.

## Data Availability

The datasets used and/or analysed during the current study available from the corresponding author on reasonable request.

## Appendix A

According to the unitary transformation, Eq. (46), the Schrödinger's equation becomes

(61) $$U_{g}HU_{g}^{†}\Phi_{g}=i\hbar \frac{\partial \Phi_{g}}{\partial t},$$

where the straightforward calculation results

(62) $$U_{g}HU_{g}^{†}=H+\frac{\hbar^{2}}{2m}\{\frac{i}{\hbar }[\frac{\partial^{2}\alpha }{\partial_{z^{2}}}+\frac{i}{\hbar }{\left(\frac{\partial \alpha }{\partial z}\right)}^{2}A_{0}]+-\frac{2}{\hbar^{2}}\frac{\partial \alpha }{\partial z}(p_{z}A_{0}-\frac{\partial \alpha }{\partial z}A_{0}^{2})\}.$$

In this calculation, the vector $A_{0}$ is assumed as a constant vector. The following derivatives will turn out to be useful for the subsequent analysis:

(63) $$\frac{\partial \alpha }{\partial z}=\frac{eE_{0}}{mc}\int \sin(\omega \eta )\;dt∼O(\frac{1}{c}),$$

(64) $$\frac{\partial^{2}\alpha }{\partial_{z}^{2}}=-\frac{eE_{0}\omega }{mc^{2}}\int \cos(\omega \eta )\;dt∼O(\frac{1}{c^{2}}).$$

All terms corresponding to the inverse square of the speed of light are omitted. The operator $UgHUg^{†}$ in the Eq. (62) may be much simplified with above relations:

(65) $$U_{g}HU_{g}^{†}=H-\frac{1}{m}\frac{\partial \alpha }{\partial z}\;p_{z}A_{0}=H+H^{\prime \prime }.$$

Obviously, because it involves the operator $p_{z}$, $H^{\prime \prime }$ leads to a transformation in the *z* direction. Considering all these, the Schrodinger equation takes the following approximation form:

(66) $$i\hbar \;\frac{\partial \Phi_{g}}{\partial t}=(H+H^{\prime \prime })\Phi_{g}.$$

We can define,

(67) $$\phi_{g}=\Omega_{g}\Phi_{g}\;,\;\Omega_{g}\equiv \exp(\frac{i}{\hbar }\int H^{\prime \prime }dt).$$

Under this transformation, Eq. (66) becomes:

(68) $$\Omega_{g}H\Omega_{g}^{†}\phi_{g}=i\hbar \;\frac{\partial \phi_{g}}{\partial t}.$$

Note that if one writes $H^{\prime \prime }=f(z)P_{z}$, then

(69) $$[f(z),p_{z}]=i\hbar \frac{\partial f}{\partial z}=i\hbar \frac{\partial }{\partial z}(-\frac{A_{0}}{m}\frac{\partial \alpha }{\partial z})=-i\hbar \frac{A_{0}}{m}\frac{\partial^{2}\alpha }{\partial z^{2}}∼O(\frac{1}{c^{2}}).$$

From this follows that $[H,\Omega_{g}]\approx 0$. Finally,

(70) $$H\phi_{g}\approx i\hbar \;\frac{\partial \phi_{g}}{\partial t},$$

and

(71) $$\Omega_{g}=\exp(\beta_{g}.\nabla ),$$

where

(72) $$\beta_{g}=-\overset{ˆ}{z}\frac{eA_{0}}{mc}\int \;\frac{\partial \alpha }{\partial z}\;dt∼O(\frac{1}{c}).$$
